# Potential Preventive Strategies for Amyotrophic Lateral Sclerosis

**DOI:** 10.3389/fnins.2020.00428

**Published:** 2020-05-26

**Authors:** B. Kuraszkiewicz, H. Goszczyńska, T. Podsiadły-Marczykowska, M. Piotrkiewicz, P. Andersen, M. Gromicho, J. Grosskreutz, M. Kuźma-Kozakiewicz, S. Petri, B. Stubbendorf, K. Szacka, H. Uysal, M. de Carvalho

**Affiliations:** ^1^Department of Methods of Brain Imaging and Functional Research of Nervous System, Nalecz Institute of Biocybernetics and Biomedical Engineering, Polish Academy of Sciences, Warsaw, Poland; ^2^Department of Clinical Sciences, Umeå University, Umeå, Sweden; ^3^Institute of Physiology, Faculty of Medicine, University of Lisbon, Lisbon, Portugal; ^4^Department of Neurology, University Hospital Jena, Jena, Germany; ^5^Jena Centre for Healthy Aging, University Hospital Jena, Jena, Germany; ^6^Department of Neurology, Medical University of Warsaw, Warsaw, Poland; ^7^Clinic for Neurology, Hannover Medical School, Hanover, Germany; ^8^Akdeniz University Faculty of Medicine, Antalya, Turkey

**Keywords:** amyotrophic lateral sclerosis, aging, dietary habits, exercise, gut microbiome, psychological stress

## Abstract

It may seem useless to propose preventive measures for a disease without established pathogenesis and successful therapy, such as amyotrophic lateral sclerosis (ALS). However, we will show that ALS shares essential molecular mechanisms with aging and that established anti-aging strategies, such as healthy diet or individually adjusted exercise, may be successfully applied to ameliorate the condition of ALS patients. These strategies might be applied for prevention if persons at ALS risk could be identified early enough. Recent research advances indicate that this may happen soon.

## Introduction

Amyotrophic lateral sclerosis (ALS) is a fatal, progressive neurodegenerative disease characterized by the loss of motoneuron function in the brain, brainstem, and spinal cord. Approximately, ALS incidence is 1–2.6 and prevalence is six per 100,000 person-years (Talbott et al., 2016), but an adult lifetime risk is estimated at 1 in 400 (Kiernan et al., 2011). The pathogenesis of ALS remains to be defined, so neither effective therapies nor preventive measures have yet been developed.

Despite the intensive research conducted in recent years, the only established risk factors for ALS remain advanced age, male gender, and certain genetic mutations (Ingre et al., 2015; Niccoli et al., 2017). Any human individual living in contemporary world is subjected to a variety of harmful environmental factors, which results in an “age-related cascade of neurodegeneration” (Drechsel et al., 2012). The effects of environmental pathogens accumulate with age (Pamphlett and Kum Jew, 2016; Bektas et al., 2018; Escobar et al., 2019; Ferrucci et al., 2020), which accelerates neurodegeneration cascade. Indeed, the age-related accumulation of heavy metals was observed in human spinal interneurons and motoneurons. Recent studies highlight several pathogenic mechanisms shared between the aging process and ALS, such as oxidative stress, metabolic deficiencies, protein aggregation, decline in mitochondrial and microglial function, and inflammation (Niccoli et al., 2017; Bektas et al., 2018; Elmore et al., 2018; Ising and Heneka, 2018). Therefore, ALS is widely considered an age-related disease (Logroscino et al., 2015; Marin et al., 2018; Pandya and Patani, 2019).

Below, we will present evidence that many anti-aging strategies may ameliorate condition of ALS patients (PALS) or decrease risk of the disease. Therefore, they could be applied also for ALS prevention. Verifying this assumption is at present impossible, since the population to which proposed procedures might be applied is indefinable. However, this situation may change in the near future due to ongoing research. In this respect, we should note the recent study of Kiernan et al. (2019), who hypothesize that adult-onset neurodegenerative conditions might have their roots in early developmental derangements and that unraveling the very early molecular events may be crucial in developing a better understanding of ALS. If this hypothesis was proven to be true, it should open the possibility to determine groups of the highest risk early enough to apply preventive strategies.

## Anti-Aging Strategies Related to ALS

### Dietary Recommendations

Literature data clearly demonstrate that dietary intervention can positively modulate the aging process and represent a prevention for many age-related diseases (Aiello et al., 2016).

The nutrients promoted as anti-aging foods (Chrysohoou and Stefanadis, 2014; Skrovankova et al., 2015) are rich in antioxidants and anti-inflammatory components, which may lower age-related risk of developing neurodegenerative diseases (Joseph et al., 2009; Yavari et al., 2015). Several phytochemicals normally present in foods, such as polyphenols, have anti-inflammatory effects on microglia (Joseph et al., 2009; Peña-Altamira et al., 2017; Fernandes et al., 2018). In particular, pomegranates contain high levels of antioxidant polyphenolic substances, as compared to other fruits and vegetables (Subash et al., 2014, 2015). It was also suggested that silibinin, a polyphenol isolated from milk thistle, exhibits neuroprotective activity by attenuating oxidative damage and astrocyte activation (Fernandes et al., 2018).

However, the studies on diet as a preventive or modifying factor for ALS are rare. A recently published big study based on the results of a multicenter American project ALS COSMOS (Nieves et al., 2016) is the first one where the associations between diet and patient’s function were evaluated in detail on the sample of 302 PALS. The results have shown that higher intakes of fiber, antioxidants, and carotenes from fruits and vegetables (“good” foods) were associated with better function, measured by ALS Functional Rating Scale Revised (ALSFRS-R) and Forced Vital Capacity scores. Few earlier studies also lend support to the use of healthy foods for prevention of this disease. A decreased ALS risk was observed in individuals whose diet was rich in fiber (Nelson et al., 2000) or vegetables and citrus fruits (Okamoto et al., 2009; Pupillo et al., 2018).

#### Foods to Avoid

The higher ALS risk was associated with increased dietary uptake of fat and glutamate (Nelson et al., 2000; Huisman et al., 2015). Also, several studies show an association between increased intake of protein from meat and aging-related diseases in elderly population [e.g., (Verburgh, 2015; Pupillo et al., 2018)]. Moreover, WHO recommendations for healthy diet^1^ postulate restriction of free sugars and trans-fats, including natural trans-fats found in meat and dairy foods from ruminant animals. Interestingly, much earlier in two papers of Patten et al. (Felmus et al., 1976; Pierce-Ruhland and Patten, 1981), the evidence that PALS drank more milk than control subjects was presented. This corresponds well to the results of Nieves et al. (2016) who indicated that “bad” foods, of which the primary component was milk, were negatively and significantly associated with ALSFRS-R score. Dairy product consumption was also found to be associated with greater risk of other age-related diseases (Grant, 1998; Hughes et al., 2017).

### Micronutrients and Supplements

Micronutrients (vitamins and minerals) play a central part in metabolism and in the maintenance of tissue function. Their influence on health, cognition, and aging is increasingly supported by experimental studies (Thomas, 2006; Hoeft et al., 2012; González-Sarríase et al., 2013). Although the amount of vitamins that are required for the proper functions of our body is relatively small, deficiency of vitamins and minerals is harmful for health (Lee et al., 2015).

Many micronutrients, such as vitamins B, C, E, D, and A have been shown to be neuroprotective, mostly because of their antioxidant properties, which is important for both anti-aging and ALS prevention (Gasperi et al., 2019; Li et al., 2019).

In the above cited study of Nieves et al. (2016), positive and significant associations with ALS function were found for selected micronutrients such as vitamins B2, B3, B6, E, K, and selenium. Vitamin E supplementation has also been shown in several other studies to correlate with lower ALS rates (Wang et al., 2011) or with better patient’s function (Patel and Hamadeh, 2009; Ngo et al., 2017). In addition, folic acid, coenzyme Q10, and melatonin were indicated as supplements holding promise to alleviate the ALS symptoms (Jacob et al., 2002; Ferrante et al., 2005; Sofic et al., 2005; Kaufmann et al., 2009; Patel and Hamadeh, 2009).

Among micronutrients, special attention has been given to vitamin D, which is known to regulate pathogenic processes involved in aging (Nagpal et al., 2005; Buell and Dawson-Hughes, 2008; DeLuca et al., 2013; Berridge, 2017). The evidence presented in the review of Hayes (2010) strongly indicates the major preventive role of vitamin D in aging.

In PALS, vitamin D blood level was proposed as a reliable prognostic factor of the disease (Karam et al., 2013; Wang et al., 2017). A severe vitamin D deficiency accelerated by four times the rate of ALSFRS-R score decline and was associated with a marked shorter life expectancy (Camu et al., 2014). Karam et al. (2013) have shown that vitamin D supplementation at 2000 international units daily resulted in a smaller decline over a period of 9 months. However, several other studies [e.g., (Yang et al., 2016; Libonati et al., 2017; Trojsi et al., 2020)] did not find any influence of vitamin D on prognosis or progression of ALS.

The most credible explanation of diverse effects of vitamin supplementation seems to be *hormesis*, which means that the individual dose–response relationship for a substance required for normal physiological function and survival is U-shaped, so that low-dose and high-dose regions have negative effects, while doses in the middle are beneficial for health (Hayes, 2007; Tuohimaa, 2009; Fricker et al., 2018). This aspect is important and should be taken into account in planning future studies. Vitamin D has been shown to be toxic in very high doses (Marcinowska-Suchowierska et al., 2018), while its deficiency has been associated with increased risk of several diseases (Holick and Chen, 2008). Moreover, it should be noted that moderate levels of reactive oxygen species (ROS) are beneficial for health and longevity (Schieber and Navdeep, 2014; Yan, 2014; Pizzino et al., 2017). Thus, antioxidant vitamins might interfere with these benefits (Lee et al., 2015), so finding the proper therapeutical dose seems crucial. However, the difficulty is to determine the proper dose for a particular person (Shenkin, 2006).

### Caloric Restriction and Gut Microbiota

Caloric restriction and fasting have long been recognized for their neuroprotective and life span-extending properties (Lanza and Nair, 2010; Lettieri-Barbato et al., 2018; Longo, 2018). Nutritional studies show that aging in animals (Calabrese et al., 2008; Kincaid and Bossy-Wetzel, 2013) and in humans (Wang et al., 2010; Chrysohoou and Stefanadis, 2014; Escobar et al., 2019) can be significantly slowed by dietary restriction.

However, in PALS, malnutrition and weight loss are commonly observed and usually associated with accelerated progression and shorter survival (Desport et al., 2000; Körner et al., 2013). Therefore, the nutritional studies in ALS focus mostly on maintenance of body weight and diets proposed for patients are usually calorie-rich (Rosenfeld and Ellis, 2008; Körner et al., 2013; Wills et al., 2014). Summing up, suggesting low-calorie diet for PALS is not appropriate.

Malnutrition in PALS may be related to diverse factors, such as difficulties in swallowing, disability that restricts access to food, or dysfunction of endogenous processes that regulate hunger, satiety, and appetite (Ngo et al., 2017, 2019). However, in about 50% of patients, the malnutrition is related to hypermetabolism, which is also linked to shorter survival (Muscaritoli et al., 2012; Ahmed et al., 2018). The causes of hypermetabolism in ALS remain undefined, although recently a new hypothesis emerged, concerning the involvement of impaired energy homeostasis in ALS pathophysiology (Ngo et al., 2015), which may be related to hypothalamic defects (Vercruysse et al., 2018).

In this respect, we should mention the recent study that revealed signs of leaky intestine and impaired microbiome in G93A-SOD1 mice (Wu et al., 2015), accompanied by an increased concentration of inflammatory cytokine IL-17A. The latter finding was also reported in a study in PALS (Fiala et al., 2010). When the intestinal microbial homeostasis was restored in these mice, gut integrity was improved and life span was prolonged (Zhang et al., 2017).

We may hypothesize that the observed discordance between presymptomatic increase in total daily energy intake and decrease in body mass index in PALS (Huisman et al., 2015; Ahmed et al., 2018) is due to the leaking intestine. If this hypothesis was proven to be true, the treatment of PALS with appropriate probiotics could restore their energy balance and allow for applying low-calorie or at least low-protein diet. Recent data confirm that dysbiosis of gut microbiota may contribute to ALS pathogenesis and progression and be a potential therapeutic target (De Marchi et al., 2018; Mazzini et al., 2018; Wright et al., 2018).

The crucial role of the gut microbiota in the host physiology and health status is being confirmed in the constantly increasing number of studies [e.g., (Kim and Jazwinski, 2018; Mangiola et al., 2018; Rothschild et al., 2018)]. The aging process deeply affects the structure of the human gut microbiota, as well as its homeostasis with the host’s immune system (Biagi et al., 2010; Dinan and Cryan, 2017; Choi et al., 2018). Age-related gut dysbiosis has also been shown to be linked to other age-related and neurodegenerative disorders (Fang, 2016; Scheperjans, 2016; Rowin et al., 2017). Therefore, supplementation with probiotics may provide novel approaches for both disease prevention and treatment (Yan and Polk, 2011; Duncan and Flint, 2014; Nagpal et al., 2018).

### Exercise

Being physically active is a key factor in maintaining health across the life span. Regular moderate-intensity training reduces oxidative stress (Webb et al., 2017; Simioni et al., 2018), decreases inflammatory markers IL-6 and CRP levels in elderly (Monteiro-Junior et al., 2017), helps to preserve cardiovascular fitness and brain function (Hotta et al., 2017; Sayegh and Degani-Costa, 2017; Shibata et al., 2018), and protects individuals from the negative effects of stress on cell aging (Puterman et al., 2010; Rebelo-Marques et al., 2018). In skeletal muscle, it attenuates mitochondrial deficits, which improves muscle function (Nyberg et al., 2012; Joseph et al., 2016; Wyckelsma et al., 2017). On the other hand, strenuous exercises generate high levels of ROS known to cause oxidative stress, activate certain pathogenic pathways, and accelerate aging (Gomez-Cabrera et al., 2009; Sahl et al., 2017).

The physical activity induces the cellular adaptations in the brain, spinal cord, and skeletal muscles that could counteract the oxidative stress complication in ALS. Therefore, it is conceivable that exercise should be beneficial for PALS (Elbasiouny and Schuster, 2011; Kincaid and Bossy-Wetzel, 2013). In G93A-SOD1 mice, moderate but not strenuous exercise delayed the onset of motor deficit (Carreras et al., 2010) and spinal motoneuron death (Deforges et al., 2009). Also, several studies in patients indicated that regular low and moderate exercise can improve functional outcome in ALS (Dalbello-Haas et al., 2008; Patel and Hamadeh, 2009; Braga et al., 2018), whereas exercises of high intensity may be harmful (Patel and Hamadeh, 2009; Wang et al., 2017).

### Telomeres

Telomeres are gene sequences present at chromosomal ends, which are responsible for maintaining genome integrity. Aging is accompanied by telomere shortening; thus, telomere length (TL) serves as a biomarker of chronological aging and an early predictor of onset of disease and increased mortality (Shay, 2018; Wang et al., 2018). For any given individual at any age, TL depends on the newborn (initial) value and the magnitude of telomere erosion from birth onward (Heidinger et al., 2012; Shalev et al., 2013).

The rate of telomere shortening may be modified by the psychosocial, environmental, and behavioral factors (Starkweather et al., 2014). All anti-aging strategies described above (such as exercise or eating foods rich in fiber and vitamins) are related to longer telomeres, whereas bad habits (such as eating processed meats and trans-fats, excessive alcohol consumption, or cigarette smoking) are related to shorter telomeres (Valdes et al., 2005; Paul, 2011; Pavanello et al., 2011). Therefore, healthy lifestyle including regular physical activity and ideal diet composition represents major preserving strategies (Seals et al., 2015).

The research on TL clearly indicates that its excessive decrease is associated with the susceptibility to age-related neurodegenerative diseases (Forero et al., 2016; Hou et al., 2019). In line with this, accelerated telomere shortening was observed in leukocytes from sporadic PALS (De Felice et al., 2014) and in ALS mice (Linkus et al., 2016). In contrast, in the recent study (Al Khleifat et al., 2019), ALS was associated with the longer telomeres. On the other hand, in the same study, the longer telomeres in patients correlated with increased survival, which would suggest that longer telomeres played a protective role. These controversies certainly call for further investigation.

### Psychological Stress

It is known that psychological stress is a major factor influencing telomere erosion (Epel et al., 2004; O’Donovan et al., 2012; Shalev et al., 2013). It is also significantly associated with lower telomerase activity and higher oxidative stress (Lavretsky and Newhouse, 2012; Mathur et al., 2016). Given that individuals who are exposed to stress during their early years show a fastest erosion rate of TL (Price et al., 2013; Savolainen et al., 2014), early intervention and prevention strategies can potentially slow down aging processes. Healthy lifestyle and environment can help to buffer the deleterious effects of stress on telomere erosion (Puterman et al., 2010; Puterman and Epel, 2012; Schutte and Malouff, 2014).

Unfortunately, the studies investigating association of ALS with psychological stress are very rare. McDonald et al. (1994) stated that psychological status is strongly related to outcome in ALS. The study of Okamoto et al. (2009) found higher ALS risk in patients reporting increased susceptibility to stress and high or moderate level of stress.

Given that PALS are subjected to enormous amount of stress, the interventions targeting resilience to it should be included in ALS modifying and preventive strategies. It should also be mentioned here that neuroprotective mechanisms can be bolstered by intellectual and physical activities.

### Therapeutic Measures Applied by Patients (ALS Reversals)

It happens very rarely that a person diagnosed with ALS stops progressing and regains significant motor function. Recently, Harrison et al. (2018) have published a study on 36 cases in whom ALS diagnosis and sustained improvement in functions were confirmed. The control group consisted of PALS without reversal, whose data were accessible from web databases. The authors reported the evident differences in the lifestyle between cases and controls. In particular, the consumption of certain supplements such as curcumin, vitamin D, or fish oil was greater for cases than for controls.

A few cases included in this study were identified from the internet^2^, where more stories of PALS claiming reversals of the disease can be found. Some of these patients have healed partially, others almost completely. Many live with the disease for 20 years or more. Most of them apply different types of detoxification (including amalgam dental filling removal). Virtually all changed their diet to consume little or no sugar, low carbohydrates and grains, and very high amounts of organic fruits and vegetables. Many of them have regular exercises, including yoga and tai-chi. Unfortunately, it is virtually impossible to confirm all their diagnoses and reversals due to the lack of authorized medical information, although the healing procedures they apply roughly correspond to those described in this review.

All those PALS changed their mental attitude to a positive one and removed or reduced most of their mental stress by applying diverse relaxation techniques, including meditations. However, most of them recall a moment of breakdown when they received the final diagnosis from a doctor and learned that there is practically nothing to be done. Only few patients were strong enough to change their attitude and decide to live with ALS instead of dying from ALS, looking for possible unconventional therapies to ameliorate their condition. The results of the study on ALS reversals (Harrison et al., 2018) prove that some of them were successful. Therefore, it is important that “A positive attitude, with an “expect-the-worst” but “prepare” and “hope-for-the-best” philosophy, is important for boosting patient morale during the management of progressive neurodegenerative diseases” (Jugdutt, 2018) should become the gold standard for providing the patient with the final diagnosis.

All the potential measures for ALS prevention and treatment are collected in Table 1, which contains references to the papers presenting clinical studies or meta-analyses with positive outcomes. Moreover, the references preceded by “IE” present indirect evidence justifying the neuroprotective action of a given procedure or nutraceutical, and those preceded by “P” contain links to PALS’ web pages.

**TABLE 1:** Potential preventive and healing procedures for ALS.

	Evidence	
	Prevention*	Treatment
Fruits and vegetables	(Nelson et al., 2000; Okamoto et al., 2009; Pupillo et al., 2018)	(Nieves et al., 2016), IE (Johnson et al., 1997; Cho et al., 2018)
Carotenoids	IE (Fitzgerald et al., 2013)	IE (Cho et al., 2018), P(Sherry, 2017)
Polyphenols	IE (Patel and Hamadeh, 2009; Fernandes et al., 2018; Rosenbohm et al., 2018)	IE (Scapagnini et al., 2011; Braidy et al., 2013; Subash et al., 2015), P (Bishop, 2014; Shackel, 2014; Sherry, 2017; Swinnard, 2018)
Curcumin	IE (Eckert et al., 2013)	(Bedlack, 2018; Chico et al., 2018; Harrison et al., 2018), IE (Calabrese et al., 2008; Scapagnini et al., 2011; Dong et al., 2014; Chico et al., 2016), P (Bishop, 2014)
Gingko biloba	IE (Mattson et al., 2002; Patel and Hamadeh, 2009)	IE (Ferrante et al., 2001; Mattson et al., 2002; Singh et al., 2019), P (Shackel, 2014; Sherry, 2017)
NRF2 activator (including luteolin and resveratrol)		(Harrison et al., 2018), IE (Wruck et al., 2007; Calabrese et al., 2008; ALSUntangled Group, 2011), P (Bishop, 2014)
Cannabidiol		(Ngo et al., 2017; Harrison et al., 2018), P (Sherry, 2017; Vivian, 2017)
Glutathione		(Harrison et al., 2018), P (ALSUntangled Group, 2018)
Coconut oil		IE (ALSUntangled Group, 2012), P (Machlan, 2012; Shackel, 2014; Sherry, 2017)
Vit. A	IE (Patel and Hamadeh, 2009; Rosenbohm et al., 2018)	(Sofic et al., 2005), P (ALSUntangled Group, 2018)
Vit. B group (B1, B3, B6, B12)	IE (Luo and Dun, 2013)	(Shen, 2011; Nieves et al., 2016), IE (Luo and Dun, 2013; Fricker et al., 2018; Gasperi et al., 2019), P (Shackel, 2014; Sherry, 2017; ALSUntangled Group, 2018)
Vit. C	(Li et al., 2019)	P (Shackel, 2014; Sherry, 2017)
Vit. D	(Camu et al., 2014; Paganoni et al., 2017; Wang et al., 2017) IE (Karam et al., 2013)	(Piquet, 2006; Ngo et al., 2017; Harrison et al., 2018), IE (ALSUntangled, 2014; Gianforcaro and Hamadeh, 2014; Lu’o’ng and Nguyễn, 2013), P (Sherry, 2017; ALSUntangled Group, 2018)
Vit. E	(Wang et al., 2011), IE (Mattson et al., 2002; Veldink et al., 2007; Patel and Hamadeh, 2009; Luo and Dun, 2013)	(Desnuelle et al., 2001; Nieves et al., 2016; Ngo et al., 2017), IE (Mattson et al., 2002), P (Sherry, 2017)
Magnesium	(Longnecker et al., 2000)	(Ngo et al., 2017), P (Machlan, 2012; Sherry, 2017; ALSUntangled Group, 2018)
Selenium		(Harrison et al., 2018), P (Shackel, 2014)
Polyunsaturated fatty acids	(Veldink et al., 2007), IE (Fitzgerald et al., 2014)	(Harrison et al., 2018), P (Shackel, 2014; Sherry, 2017)
Melatonin	(Sofic et al., 2005), IE (Patel and Hamadeh, 2009; Lee et al., 2020)	IE (Iacovitti et al., 1997; Jacob et al., 2002), P (Sherry, 2017)
Q10	IE (Patel and Hamadeh, 2009)	(Jacob et al., 2002; Sofic et al., 2005; Ngo et al., 2017), IE (Ferrante et al., 2005), P (Shackel, 2014; Sherry, 2017)
Folic acid	IE (Mattson et al., 2002; Patel and Hamadeh, 2009)	IE (Mattson et al., 2002; Luo and Dun, 2013)
Azathioprine (immunosuppressive medication)		(Harrison et al., 2018), IE (Malaspina et al., 2014), P (Sherry, 2017)
Gut microbiome, probiotics	IE (De Marchi et al., 2018; Mazzini et al., 2018; Wright et al., 2018), IE (Duncan and Flint, 2014; Dinan and Cryan, 2017; Sun, 2017; Choi et al., 2018; Erber et al., 2019)	(Huisman et al., 2015; De Marchi et al., 2018; Mazzini et al., 2018), IE (Biagi et al., 2010; Fiala et al., 2010; Sun, 2017; Wright et al., 2018), P (Sherry, 2017)
Calorie restriction, fasting		P (Bishop, 2014; ALSUntangled Group, 2018)
Liver cleansing diet		P (Shackel, 2014; Sherry, 2017; Swinnard, 2018)
Ketogenic, paleolithic, and high-fat diets		(Ngo et al., 2017),IE (Zhao et al., 2006), P (Coelho, 2015)
Gluten-free diet		P (Cherry, 2017)
Detoxification of the environment and/or the body (including removal of amalgam fillings)		P (Shackel, 2014; Healing ALS Team, 2017b; Sherry, 2017; ALSUntangled Group, 2018; Swinnard, 2018)
Regular low and moderate intensity exercise	(Hamidou et al., 2014; Wang et al., 2017), IE (Patel and Hamadeh, 2009; Elbasiouny and Schuster, 2011; Kincaid and Bossy-Wetzel, 2013; Lisle and Tennison, 2015; Seals et al., 2015; Rosenbohm et al., 2018)	(Braga et al., 2018), IE (Deforges et al., 2009; Patel and Hamadeh, 2009), P (Bishop, 2014; Shackel, 2014; Coelho, 2015; Cherry, 2017; Swinnard, 2018)
Physiotherapy, yoga, tai-chi		(Ribeiro, 2014), IE (Jugdutt, 2018), P (Shackel, 2014)
Oxygen therapy with exercise or hyperbaric		P (Cherry, 2017; Vivian, 2017)
Chelation therapy	IE (Patel and Hamadeh, 2009; Rosenbohm et al., 2018)	P (Machlan, 2012; Healing ALS Team, 2017a; Sherry, 2017)
Holistic treatments: energy healing, Chinese medicine, chiropractice, homeopathy		(Pan et al., 2013), P (McDonald, 1988; Bishop, 2014; Buss, 2016; Healing ALS Team, 2017b)
Positive mental attitude, avoidance of any physical and mental stress, increased self-awareness, and serious emotional good changes	(McDonald et al., 1994; Okamoto et al., 2009; Puterman et al., 2010; Puterman and Epel, 2012; Schutte and Malouff, 2014)	(Harrison et al., 2018), P (McDonald, 1988; Cherry, 2017; Healing ALS Team, 2017b; ALSUntangled Group, 2018)
Psychotherapy, relaxation technique, meditation		P (McDonald, 1988; Buss, 2016; Healing ALS Team, 2017b; Sherry, 2017)
Prayer		P (Bishop, 2014; Coelho, 2015; Moore, 2019)

*Studies presenting evidence that the given factor was associated with a reduced risk of ALS. IE, Indirect evidence (studies in AD and PD and aging, animal and in vitro studies, reviews, theoretical considerations based on neuroprotective effects of given procedure or nutrient). P, stories from patients claiming ALS reversal for more than 5 years. NRF2, nuclear factor erythroid-2-related factor 2 is a transcription factor that activates over 500 genes via molecules called sirtuins.

## Summary and Discussion

The anti-aging strategies have been shown in several studies to decrease ALS risk or ameliorate PALS condition. Although there are other studies that did not find such effects, the lack of an evidence is not the evidence of nonexistence. Therefore, in this mini-review, we have concentrated on the positive results. Especially encouraging are stories of patients with reversals, which should be the object of future research efforts focused on establishing the most effective lifestyle practices for maintaining function in ALS (Seals et al., 2015). Moreover, the doses optimal for maximum effects will have to be determined for personalized therapeutic and/or preventive interventions.

It has been shown that the early-life diet plays an essential role in the individual’s health status and longevity, as well as the later development of aging-related chronic diseases (Vaiserman, 2014). Also, the telomeres are the most susceptible to erosion in the first years of life (Price et al., 2013). Therefore, applying healthy lifestyle from the earliest stages of human development is very important. There is an urgent need for actions that would popularize such lifestyle among the wide public, which might prolong the health span, decreasing the risk of ALS and many other age-related diseases. This is an extremely difficult task, but not impossible. The recent study in a group of adolescents has proved that the special psychological intervention, presenting unhealthy dietary choices as incompatible with important values, can change youngsters’ dietary attitudes toward healthy food (Bryan et al., 2019).

## Author Contributions

MP wrote the draft. MP, BK, HG, TP-M, participated in collection of the references. MdC supervised the project. All the authors participated in discussions leading to the final version of the manuscript and accepted this version for publication.

## Conflict of Interest

The Reviewer DSF declared a past co-authorship with authors SP and JG to the handling Editor.

The remaining authors declare that the research was conducted in the absence of any commercial or financial relationships that could be construed as a potential conflict of interest.

## Footnotes

1 https://www.who.int/news-room/fact-sheets/detail/healthy-diet

2 https://healingals.org/ and https://www.alswinners.com/
